# Optimization, characterization, and cytotoxicity studies of novel anti-tubercular agent-loaded liposomal vesicles

**DOI:** 10.1038/s41598-023-49576-2

**Published:** 2024-01-04

**Authors:** Manar M. Obiedallah, Maxim A. Mironov, Danila V. Belyaev, Antoaneta Ene, Diana V. Vakhrusheva, Svetlana Yu. Krasnoborova, Sergey Y. Bershitsky, Daniil V. Shchepkin, Artem S. Minin, Rashida I. Ishmetova, Nina K. Ignatenko, Svetlana G. Tolshchina, Olga V. Fedorova, Gennady L. Rusinov

**Affiliations:** 1 Institute of Chemical Technology, Ural Federal University, Yekaterinburg, Russia; 2 Department of Pharmaceutics, Faculty of Pharmacy, Assiut University, Assiut, 71526 Egypt; 3 I. Postovsky Institute of Organic Synthesis, Ural Branch of the Russian Academy of Sciences, S. Kovalevskaya Str. 22, Yekaterinburg, 620108 Russia; 4 National Medical Research Center of Phthisiopulmonology and Infectious Diseases, 22 Parts’ezda St., 50, Yekaterinburg, 620039 Russia; 5 INPOLDE Research Center, Department of Chemistry, Physics and Environment, Faculty of Sciences and Environment, Dunarea de Jos University of Galati, 47 Domneasca Street, 800008 Galati, Romania; 6 Institute of Immunology and Physiology, Ural Branch of Russian Academy of Sciences, Yekaterinburg, 620049 Russia; 7 Institute of Natural Sciences and Mathematics, Ural Federal University, Yekaterinburg, Russia; 8 M.N. Mikheev Institute of Metal Physics of the Ural Branch of the Russian Academy of Sciences, S.Kovalevskaya St. 18, Yekaterinburg, 620108 Russia

**Keywords:** Drug delivery, Biotechnology, Drug delivery, Pharmaceutics

## Abstract

The treatment of tuberculosis is still a challenging process due to the widespread of pathogen strains resistant to antibacterial drugs, as well as the undesirable effects of anti-tuberculosis therapy. Hence, the development of safe and effective new anti-antitubercular agents, in addition to suitable nanocarrier systems, has become of utmost importance and necessity. Our research aims to develop liposomal vesicles that contain newly synthesized compounds with antimycobacterial action. The compound being studied is a derivative of imidazo-tetrazine named 3-(3,5-dimethylpyrazole-1-yl)-6-(isopropylthio) imidazo [1,2-b] [1,2,4,5] tetrazine compound. Several factors that affect liposomal characteristics were studied. The maximum encapsulation efficiency was 53.62 ± 0.09. The selected liposomal formulation T8* possessed a mean particle size of about 205.3 ± 3.94 nm with PDI 0.282, and zeta potential was + 36.37 ± 0.49 mv. The results of the in vitro release study indicated that the solubility of compound I was increased by its incorporation in liposomes. The free compound and liposomal preparation showed antimycobacterial activity against *Mycobacterium tuberculosis* H_37_R_v_ (ATCC 27294) at MIC value 0.94–1.88 μg/ml. We predict that the liposomes may be a good candidate for delivering new antitubercular drugs.

## Introduction

Almost a quarter of the world's population is affected by pulmonary tuberculosis, a prevalent respiratory disease^1^. *Mycobacterium tuberculosis (M. tuberculosis*) invades and multiplies within macrophages, rendering them typically inaccessible to anti-tuberculosis medications. Although the oral and parenteral routes of administration are successful for treating tuberculosis, a number of studies have revealed undesirable side effects that have led to treatment discontinuation^2^. Anti-tuberculosis medication therapy necessitates long-term high dose administration, whereas only a limited quantity of the medication reaches the infection site. As a result, some medication molecules may persist in the body and cause serious side effects. Rifampicin, for instance, when taken orally, may result in nausea, flu-like symptoms, abrupt renal failure, hepatotoxicity, and agranulocytosis^3^.

Moreover, taking isoniazid orally has been linked to the cessation of treatment due to convulsive seizures, mental impairment, coma, vasculitis, polyneuritis, and hepatotoxicity^4^. The second most common challenge that occurs during the treatment of tuberculosis is the multi-drug resistance (MDR) of mycobacteria to anti-tuberculosis drugs, which leads to treatment failure. In order to improve the clinical outcome and reduce side effects, new antitubercular (anti-TB) drugs, as well as lipid and polymer-based nanoparticle drug delivery systems, have been developed and investigated. One of the lipid-based nanocarrier systems is liposomes. Kósa et al. studied the efficacy of two novel antitubercular agents candidates in free and liposome-encapsulated formulations^5^. Zhao et al. have prepared and evaluated paclitaxel liposomes in vivo for lung targeting delivery in dogs^6^.

In comparison to paclitaxel injection, paclitaxel liposomes demonstrated superior lung-targeting properties. Recent years have seen a rise in the usage of liposomes for the treatment of pulmonary disorders, due to their formulation from phospholipids similar to biological pulmonary surfactant, which gives them unique properties for pulmonary medication administration^7^. Vyas et al. developed ligand-anchored liposomal aerosols to enhance rifampicin delivery to alveolar macrophages^8^. In contrast to the free drug, the ligand-anchored liposomes were capable of efficiently achieving high drug concentrations in alveolar macrophages and retaining those concentrations for an extended period of time^8^.

Deol and Khuller also improved TB chemotherapy by using lung-specific modified liposomes^9^. Inhalational TB treatment with pyrazinamide-loaded proliposomes has been recommended by another study^10^. It was found that pyrazinamide proliposomes were less hazardous to kidney, liver, and respiratory-associated cells. Inhalable ciprofloxacin nanocrystals encapsulated in liposomal powders were created by Khatib et al.^11^. Liquid liposomal formulations were freeze-thawed and then spray-dried with sucrose as a lyoprotectant, resulting in the desired formulations. These liposomal powders demonstrated both great respirability and physical stability upon exposure to moisture and sustained drug release over an extended period of time^11^.

**Figure 1:**
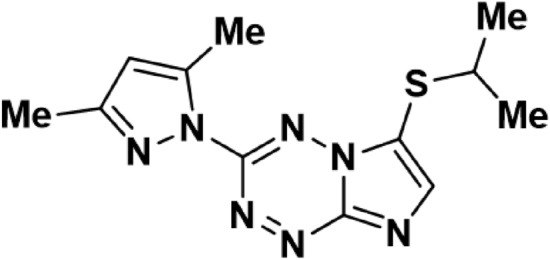
Chemical structure of 3-(3,5-dimethylpyrazole-1-yl)-6-(isopropylthio) imidazo [1,2-b] [1,2,4,5] tetrazine I (compound I).

Our research focuses on a new antimycobacterial agent, which was synthesized by the Institute of Organic Synthesis, Ural Branch of the Russian Academy of Sciences. The compound name is 3-(3,5-dimethylpyrazole-1-yl)-6-(isopropylthio) imidazo [1,2-b] [1,2,4,5] tetrazine (Fig. 1), which referred to as compound I in our article. Compound I is an imidazo-tetrazine derivative. The compound showed antimycobacterial activity on both *Mycobacterium tuberculosis H*_*37*_*R*_*v*_ and *Mycobacterium smegmatis mc2 155*, with a concentration as low as 1 μg/ml^12^. The median lethal dose (LD50) of imidazo [1,2-b][1,2,4,5] tetrazines in mice was reported to be 499 mg/kg^13^. However, the results of the solubility study showed that compound I is slightly soluble in water (solubility in water 0.44 mg/ml). The aim of this research was to develop and characterize liposomal vesicles for the effective delivery of a newly synthesized antimycobacterial compound I, a derivative of imidazo-tetrazine, to improve the treatment of tuberculosis. This study focused on enhancing the solubility, evaluation of the cytotoxicity, and investigation of the antimycobacterial activity of the compound I through encapsulation in liposomes, particularly in the face of rising drug-resistant strains and the limitations of current treatments.

## Materials and methods

### Materials

The antimycobacterial compound 3-(3,5-dimethylpyrazole-1-yl)-6-(isopropylthio) imidazo [1,2-b] [1,2,4,5] tetrazine (compound I, Fig. 1) was synthesized by the Institute of Organic Synthesis, Ural Branch of the Russian Academy of Sciences. The phospholipids, Epikuron 200, composed of phosphatidylcholine enriched fraction of soybean lecithin (PC), was obtained from Cargill Inc. (Germany). Cholesterol (CL) was purchased from Alfa Aesar company (Belgium). Choline palmitic acid ester (CPA) (positive charge generating agent) was synthesized in the laboratory according to specific protocol. Tween 80 and DMSO were purchased from Bekton company (Russia). Methyl ester of Palmitic acid (MPA) was synthesized in the laboratory. Dichloromethane, chloroform, and ethanol were purchased from Acros (Russian Federation). Dialysis membrane with molecular weight cut-off of 6–8 kDa from Orange Scientific company (Belgium). Middlebrook 7H9 nutrient broth was from BD DIFCO company (USA), OADC (BD BBL MGIT OADC) as a growth additive, resazurin solution purchased from (China). Full Dulbecco's Modified Eagle nutrient medium (DMEM) with L-glutamine, 10% embryonic veal serum, was from PanEco company (Russia). Gentamicin was from Biolot company (Russia). Trypsin and Versen from Biolot (Russia). MTT solution was from DIA-M company (Russia).

### Synthetic structure, characterization, and physicochemical properties of compound I

The antimycobacterial compound; 3-(3,5-dimethylpyrazole-1-yl)-6-(isopropylthio) imidazo [1,2-b] [1,2,4,5] tetrazine I was synthesized starting from imidazo[1,2-b][1,2,4,5] tetrazine I (Figure M1, Supplementary Materials). To a solution of 108 mg (0.5 mmol) of imidazo [1,2-b][1,2,4,5] tetrazine I in 5 ml of acetonitrile, 46 mg (0.6 mmol) of mercaptan and 50 mg (0.5 mmol) of triethylamine were added and then stirred for 7 h at room temperature (TLC control). Afterwards, the solvent evaporated. Compound I was isolated by column chromatography using benzene and acetonitrile eluent in a ratio of 1:1. Compound I is a crystalline yellow powder, soluble in ethanol, acetonitrile, dichloromethane, and chloroform. The melting point of compound I is 108–109 °C. LC/MS (ESI): Found m/z = 290.1184 (M−). C12H16N7S+. Calculated 290.1182 ([M+H]+) (Figure M2, Supplementary Materials).

The structure of compound I was elucidated by ^1^H nuclear magnetic resonance spectrometry (^1^H NMR) on an Avance DRX-400 spectrometer (Bruker BioSpin, Germany) with an operating frequency of 400 MHz using TMS as an internal standard. The purity of compound I was evaluated by gas chromatograph (GC) Agilent 7890A equipped with mass-spectrometer 5975 inert XL (Agilent Technologies, USA). In addition, the high-resolution mass spectra analysis (HRMS) was performed using a LC-MS/MS Q-TOF system (Bruker maXis impact HD Q-TOF with Agilent 1260 series LC). IR spectra were recorded on a Nicolet 6700 IR Fourier spectrometer (Intertech Corporation, USA). The absorption spectrum of compound I was recorded on a UV–Vis spectrophotometer (2401 PC, Shimadzu Corporation, Japan).

### Solubility studies of compound I in water and at different pH

The solubility of the compound I in water and at different pH (1.2, 6.8) was determined by the shake flask method. Briefly, an excess amount of compound I was added to 10 ml of the following aqueous solutions; distilled water, 0.1 N HCl (pH 1.2), and phosphate buffer solution (pH 6.8) in perfectly closed glass conical flasks. The samples were covered with foil paper and placed in a shaker water bath at 37ºC 24 h. These samples were then filtered through a 0.2 µm syringe filter (Whatman Anotop 25), diluted, and analyzed for drug content by UV–Vis spectrophotometer at λ_max_ 256 nm.

### Preparation of plain liposomes and compound I loaded liposomes

The standard lipid thin film hydration process was used to prepare liposomes^14,15^. Briefly, the lipid components PC:CL with different weight ratios, steric stabilizing agent (MPA or Tween 80), and charge forming agent (CPA) were dissolved in the organic solvent. For the preparation of compound I loaded liposomes, the definite amount of compound I was also dissolved in the organic solvent. The total weight of ingredients in addition to the compound I dissolved in the organic solvent, is 200 mg. Tween 80 and methyl ester of palmitic acid were evaluated as steric stabilizing agents^16^. The organic solvent was then evaporated using a rotary evaporator (Model HB 10 D SP9, IKA) at a temperature of 43 °C for 1 h. A desiccator was used to further dry the thin lipid film. Different aqueous solutions (phosphate buffer (pH 7.4), 0.9% NaCl solution, and distilled water) were investigated for hydration of the lipid layer. The aqueous solution that produced the most stable liposomal suspension was selected for the liposomal preparation. The sample was then extruded two times through a 0.2 µm syringe filter at 50 °C to obtain small unilamellar liposomes. The liposomal suspensions were then stored in the refrigerator at 4ºC for further studies. The physical stability of each liposomal suspension was checked visually after one day of storage at 4 °C before further studies.

### Optimization of prepared liposomes loaded with compound I

Different factors that affect the characteristics of the prepared liposomes loaded with compound I were studied and evaluated. These factors are the type and volume of organic solvent, different concentrations of antimycobacterial agent, type and concentration of steric stabilizing agent, and the effect of different types of hydrating aqueous solutions. All these variables are comprehensively examined in the “Results and Discussion” section.

### Characterization of liposomal preparations loaded with compound I

#### Particle size, polydispersity index (PDI), and zeta potential determinations

The particle size, PDI, and surface charge (Zeta potential) were measured by dynamic light scattering technique using Zetasizer Nano SL (Malvern Instrument, U.K.) at 25°C. The measurements were performed after diluting the samples five-fold with distilled water at ambient temperature. Each sample was measured in triplicate, and the average value was calculated^17^.

#### Entrapment efficiency determination

The amount of entrapped drug in liposomes was determined using a UV–Vis spectrophotometer by the indirect method^18,19^. Free compound I was separated from compound I encapsulated liposomes by centrifugation at (28,000 rpm, 51,714 × g) in a refrigerated centrifuge (Biofuge 28RS, Heraeus Sepatech, USA) for 1 h. A volume of 200 µl of the supernatant was withdrawn, diluted with distilled water, and the absorbance was measured spectrophotometrically at λ_max_ 256 nm. The concentration of free compound I was calculated using the equation obtained from the calibration curve of compound I in distilled water. The amount of entrapped compound I inside the liposomes was calculated by subtraction of the free amount of compound I in supernatant from the initial or total amount of compound I added in the preparation of liposomes according to the following Eq. (1), (EE%);

1 $$EE\% = \frac{Total \;amount\; of \;compound \;I\; in \;liposomes - Free\; amount\; of \;compound\; I \;in \;supernatent}{{Total\; amount\; of\; compound\; I \;in \;liposomes}} \times 100$$

#### In vitro release study

The dialysis bag diffusion method was used for the in vitro drug release investigation. The membrane's molecular weight cutoff was 6–8 kDa. The bag was soaked in phosphate buffer solution (PBS) (pH 6.8) overnight before use. Definite volumes of the selected liposomal preparation T8 * (equivalent to 1.25 mg of pure compound I) and pure compound I were placed into dialysis bags with the two ends fixed by clips. The bags were placed in beakers filled with 50 mL of PBS (pH 6.8). The beakers were placed into a multifunctional water bath shaker (Model SB-22) at 37 °C and 100 rpm. At different time intervals (0.25, 0.5, 1, 2, 3, 4, 5, 6, and 12 h), 1 ml was taken from the release medium and replaced with the same volume of fresh medium in order to keep the sink condition. The withdrawn volumes were diluted with PBS, and the absorbance was measured spectrophotometrically using UV–Vis spectrophotometer at λ_max_ 256 nm against blank sample treated similarly. The concentration of the released compound I was calculated using calibration curve of compound I in PBS (pH 6.8). All experiments were repeated three times, and the results were expressed as means ± standard deviation.

#### Atomic force microscopy (AFM)

The measurements were conducted using an NTEGRA Therma scanning probe microscope (NT-MDT, Zelenograd, Russia) equipped with NSG01 silicon probes. The surface topography of the particle was assessed using atomic force microscopy in semi-contact mode. The particles were put onto a cover slip, then a 5 μl droplet was applied onto a substrate and allowed to air-dry. The scanning probe microscope detected the existence of residual traces of a "buffer solution" on the surface where the particles were found. Consequently, the specimen underwent a cleansing process involving deionized water. Specifically, a droplet of deionized water was carefully applied onto the substrate, focusing on the region where the particles were located. After 1 min, the water was subsequently eliminated using filter paper, and the specimen was dried using compressed air.

### Biological studies

#### In vitro antimycobacterial activity

All biological studies were performed and approved by the Ural Research Institute of Phthisiopulmonology (Ekaterinburg, Russia). The antitubercular activity of empty liposomes, compound I loaded liposomes (sample T8*), and pure compound I was determined using resazurin microtiter assay plate (REMA) as thoroughly described^20,21^ REMA assay was performed in 96-well flat bottom plates^22^. A suspension of *Mycobacterium tuberculosis* with an optical density of 1.0 McFarland units was prepared (using saline) from a culture of *M. tuberculosis H*_*37*_*R*_*v*_ (ATCC 27294) inoculated in Lowenstein-Jensen nutrient medium and located in the logarithmic growth phase. After that, 50 μl of the resulting suspension was transferred to a test tube with Middlebrook 7H9 nutrient broth supplemented with OADC as a growth additive. The amount of 10 mg of pure compound I was dissolved in 1.0 ml of DMSO to obtain a base solution with a concentration of 10,000 μg/ml. Further dilutions of the base solution with DMSO were carried out to obtain different solutions with serial concentrations of 1000, 500, 250, 125, 62.5, 31.25, 15.63, and 7.81 μg/ml were introduced into the wells of the plates. A volume of 97 μl of the nutrient medium was added to the wells of the plate, then 3 μl of each solution with the previously prepared serial concentrations of the compound I and 100 μl of MBT suspension were introduced into the wells of the plates to obtain the following concentrations of the test compound: 15, 7.5, 3.75, 1.88, 0.94, 0.47, 0.23 and 0.12 μg/ml (DMSO concentration—1.5% v/v).

For the preparation of the solutions with different dilutions of sample T8* (concentration of compound I in sample T8* was 500 μg/mg), sterile distilled water was used as a diluent to obtain the following concentrations of the compound I in liposomal suspension: 300, 150, 75, 37.5, 18.75, 9.38, 4.69 and 2.34 μg/ml. After that, a volume of 90 μl of the nutrient medium and 10 μl of each solution of the sample T8* were introduced into the wells of the plates. Further, 100 μl of MBT suspension was introduced into the wells of the plates as previously described with pure compound I to obtain the following concentrations: 15, 7.5, 3.75, 1.88, 0.94, 0.47, 0.23, and 0.12 μg/ml.

In order to evaluate the effect of DMSO, sample T8* was prepared by the addition of 87 μl of nutrient medium, 3 μl DMSO, and 10 μl of each solution of the previously prepared different concentrations of sample T8* into the 96-wells of the plates. As explained above, 100 μl of MBT suspension were introduced into the wells to obtain the following concentrations of the liposomal suspension T8*: 15, 7.5, 3.75, 1.88, 0.94, 0.47, 0.23 and 0.12 μg/ml (DMSO concentration 1.5% v/v). The empty liposomal solution was prepared as described with the sample T8* but without DMSO, and the concentrations of 15 and 7.5 μg/ml were obtained in the wells of the plate. The plates were incubated at 37 °C for 7 days. At the end of the incubation time, 30 μl of resazurin solution was introduced into the wells with the addition of Tween 80, and incubation continued at 37 °C. The results were taken after 24, 48 and 72 h. The minimum inhibitory concentration (MIC) was defined as the lowest concentration of compound I that prevented a color change of resazurin^22^.

#### Cell cultures and cytotoxic evaluation

The cytotoxicity of empty liposomes, compound I loaded liposomes (sample T8*) and pure compound I at different concentrations on the Vero cell line (ATCC CCL-81) was investigated by MTT assay^23^. The transplantable Vero cell line, obtained from the epithelium of a kidney taken from the African green monkey (Chlorocebus aethiops), was used in the work; the permission of the ethical committee for its use is not required. Primary cell lines were not used in the work. The Vero cell line (ATCC CCL-81) was incubated in a DMEM with L-glutamine containing 10% embryonic veal serum and 50 μg/ml of gentamicin in a CO_2_ incubator (Binder, Germany) at 37 °C. Upon reaching the monolayer in the culture flask (Jet Biofil, China), the cells were treated with a warm solution of trypsin: versen (1: 1 ratio), the culture was then transferred from the monolayer to the suspension and the resulting suspension was centrifuged. The supernatant was removed, the precipitate was resuspended in 1 ml of the full nutrient medium, and the cells were counted using the Goryaev's chamber. Based on the value obtained, a suspension with a content of 15,000 cells/ml in a full feeding medium was prepared. A volume of 200 μl of the resulting suspension (3000 cells/ well) was introduced into the wells of the 96-well plate. The plates were incubated for 24 h in a CO_2_ incubator at 37 ^º^ C. Different dilutions of pure compound I (using DMSO as diluent) and sample T8* (using distilled water as diluent) were prepared to obtain the following concentrations: 3000, 750, 187.5 and 46.88 μg/ml for pure compound I, and 300, 75, 18.75 and 4.68 μg/ml for sample T8*.

Further dilutions with full nutrient medium were carried out to obtain the following concentrations: 15, 3.75, 0.94, and 0.23 μg/ml for both pure compound I (conc. of DMSO was 0.5% v/v) and sample T8*. Solutions of "empty" liposomes were prepared similarly to solutions of the sample T8*. At the end of the 24-h incubation, the nutrient medium was removed from the wells of the plates, and 200 μl of the previously prepared solutions of the compound I, sample T8*, and the empty liposomes were introduced into each well of the plates. Intact control wells were filled with 200 µl of full nutrient medium. Further 200 µl of full nutrient medium supplemented with DMSO (final concentration in well 0.5% v/v) was added to wells with DMSO. The plates were incubated for 24 h in a CO_2_ incubator at 37 °C. After that, the nutrient medium with substances was removed. Then a volume of 200 μl of fresh full nutrient medium and 50 μl of an aqueous solution of MTT reagent (5 mg/ml) were introduced into each well. The plates were incubated for another 4 h in CO_2_ incubator at 37° C, then, the nutrient medium was removed, a volume of 200 μl of DMSO and 30 μl of glycine buffer were introduced. The plates were shaken to dissolve the formazan crystals for 10 min. The optical density of the solution in each well was measured at 540 nm using a flatbed spectrophotometer (Multiskan FC, Thermo Scientific). IC50 was calculated as the compound concentration causing a decrease in cell viability by 50%.

### Statistical analysis

Each test was done in triplicate, and the data was expressed as means ± SD. The statistical evaluation of differences between the results was carried out by the one-way analysis of variance test and Tukey post-test using Origin pro 2021 software. The significance of coefficients was presented by, *p < 0.05; **p < 0.01 or, ***p < 0.001. The obtained biological data was processed using Microsoft Office Excel 16 and GraphPadPrism v.8.0.1 software using non-linear regression analysis.

## Results and discussion

### Characterization of compound I

**Figure 2:**
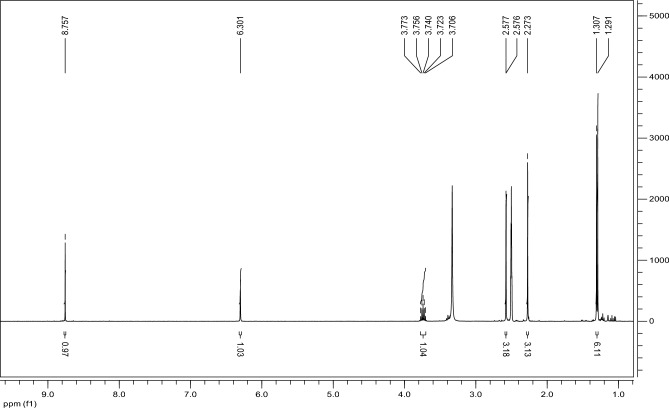
^1^H NMR spectra of compound I.

In order to understand the chemical structure and characteristics of compound I, several analytical techniques were employed and investigated.The ^1^H NMR spectrum of compound I (Fig. 2) showed signals of protons of the isopropyl group (doublet and multiplet in the δ region of 1.30 and 3.74 ppm, respectively), pyrazole substituent (singlets in the δ region of 2.27, 2.58 and 6.30 ppm), as well as a singlet at δ of 8.76 ppm, related to the H (7) proton in the imidazotetrazine ring.

**Figure 3:**
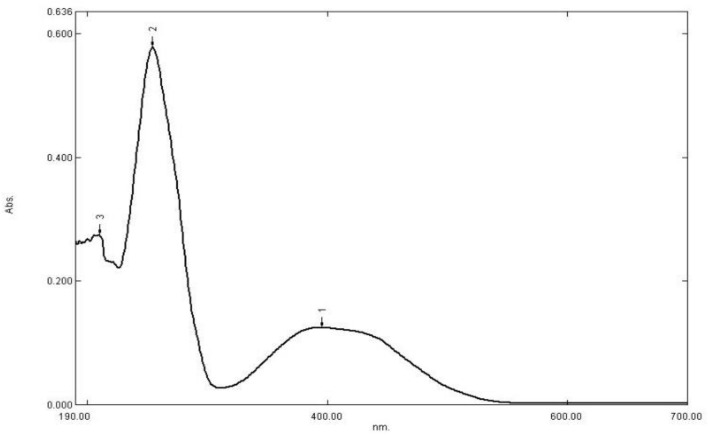
UV spectrum of compound I in acetonitrile.

No impurities were recorded by the GC analysis, the retention time of the compound I was found to be 22.0 min (Fig. M3, Supplementary Materials). In the IR spectrum of compound I (Fig. M4, Supplementary Materials), the weak absorption bands at 2964, 2929, and 2865 cm^−1^ belong to antisymmetric and symmetrical valence oscillations of C–H bonds of methyl groups. The absorption band of low intensity with a frequency of 3110 cm^−1^ is related to the valence oscillations of methine protons of pyrazole and imidazole fragments. In a wide range of 1600–1100 cm^−1^, strict assignment of absorption bands is difficult due to the manifestation of valence oscillations of the pyrazole and imidazole rings in this region, as well as deformation oscillations of C-H bonds of methyl substituents. The intense absorption band at 1058 cm^−1^ can be attributed to the valence oscillations of the tetrazine ring. The UV spectrum of compound I (Fig. 3) shows that it has two characteristic peaks in the UV region. One broad band at λ_max_ 395 nm and the other sharp peak at λ_max_ at 256 nm. We selected the second sharp peak at λ_max_ at 256 to perform calibration curves as it is more sensitive.

### Solubility of the compound I in water and in different pH

An essential physicochemical characteristic of a drug's molecule is its solubility, which directly impacts the process of discovering and developing new drugs^24^. The solubility of compound I was found to be 0.44, 0.083, and 0.308 mg/ml in distilled water, 0.1 N HCl (pH 1.2), and phosphate buffer solution (pH 6.8) respectively. This indicated that compound I is slightly soluble in water.

### Optimization of liposomes loaded with compound I

In the first optimization process, we used different amounts of methyl ester of palmitic acid (5, 10 mg) as a steric stabilizing agent and choline ester of palmitic acid (5, 10, 15 mg). Visual assessment was conducted one day after storage to assess the physical instability of liposomal formulations that exhibited aggregation or precipitate, resulting in the development of turbidity. The formulations that have shown turbidity or precipitation were excluded from further studies. The stability of the liposomal preparations may be due to the optimal concentration of the stabilizer (methyl ester of palmitic acid) on the surface of liposomes. Table 1 demonstrates the composition of various liposomal preparations with different ratios of lipid components. From the results of the physical stability that are shown in Table 1, we selected the ratios of lipid components used in S5 preparation as the best ratios for further studies.

**Table 1:** Composition of different liposomal preparations loaded and unloaded with compound I.

Batch no.	PC:CL (mg)	CPA (mg)	MPA (mg)	compound I (mg)	Physical stability after 1 day
S1	180: 10	5	5	–	Unstable
S2	175: 10	5	10	–	Unstable
S3	175: 10	10	5	–	Unstable
S4	170: 10	15	5	–	Stable
S5	165: 10	15	5	5	Stable
S6	155: 20	15	5	5	Unstable
S7	145: 30	15	5	5	Unstable

The organic solvent used for all formulations is chloroform; 20 ml. The hydration media was phosphate buffer (pH 7.4).

#### Type and volume of organic solvent

Despite the fact that S4 and S5 were stable, the entrapment efficiency (EE%) of S5 was less than 10%. We tried to modify different formulation parameters, such as the volume and type of organic solvent and drug concentration, to enhance the entrapment efficiency. Three different types of organic solvents were used (ethanol, chloroform, and dichloromethane (DCM), with three different volumes (5, 10, and 20 ml). The effect of the type and volume of organic solvent on particle size and entrapment efficiency was evaluated. Table 2 shows the different liposomal samples (S8-S13) prepared using different types and volumes of organic solvent. Formulations (S8-S13) possessed the same amounts as in formulation S5 but with different types and volumes of organic solvent.

**Table 2:** Liposomal samples (S8–S13) prepared with different types and volumes of organic solvent.

Sample	Ethanol (ml)	Chloroform (ml)	DCM (ml)
S8	5	–	–
S9	10	–	–
S10	–	5	–
S11	–	10	–
S12	–	–	5
S13	–	–	10

The composition of all formulations (S8–S13) is the same as the composition of S5 formula but with different types and volumes of organic solvent.

**Figure 4:**
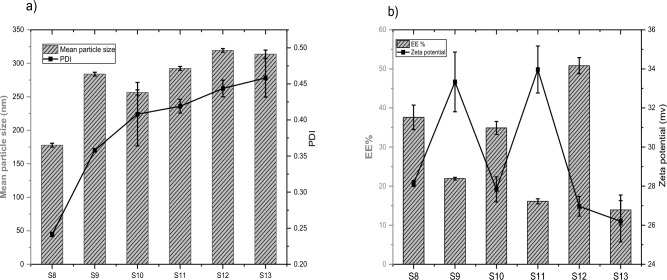
**(a, b)** The mean particle size, PDI and EE%, zeta potential of liposomal formulations (S8–S13) with different types and volumes of organic solvents (n = 3).

The effect of the type and volume of organic solvent on the characteristics of the liposomal preparation are demonstrated in Fig. 4a, b. Using different types and volumes of organic solvent significantly affects the mean particle size and EE%. The mean particle size ranged from 177.7 ± 2.8 nm using 5 ml of ethanol to 319.3 ± 2.75 nm when using 5 ml of DCM. It was observed that the samples prepared with DCM were unstable, and aggregation occurred, which may interpret the significant increase in the mean particle size of samples S12 and S13 compared to S8-S11 (P < 0.05). The PDI ranged from 0.242 for sample S8 to 0.458 for sample S13. The PDI was significantly different between S8, S9; S8, S10; S8, S11; S9, S11; S8, S12; S9, S12; S8, S13 and S9, S13 (P < 0.05). This agrees with López et al., who reported that the type of organic solvent influence liposome characteristics (size, PDI, and zeta potential)^25^. The authors interpreted these results in the light of the relationship between the solvent polarity and average liposome size^25^. The results of EE% demonstrated a significant increase in the percentage of entrapped drug by decreasing the volume of organic solvent from 10 to 5 ml for all types of the organic solvents used (P < 0.0001), Fig. 4b. In the case of using 5 ml ethanol (S8) or 5 ml chloroform (S10), the EE% was 37.6 ± 3.14 and 34.9 ± 1.65 respectively. However, there was no significant difference between samples S8 and S10 (P > 0.05). On the other hand, the EE% was significantly decreased to 21.92 ± 0.34 and 16.13 ± 0.64 for samples S9 and S11 (10 ml of ethanol and chloroform) compared to S8 and S10 (P < 0.001). The EE% was 13.9 ± 3.82 for sample S13 (prepared using 10 ml DCM); however, it reached 50.82 ± 2.09 when using 5 ml DCM (sample S12). The zeta potential was from 26.2 ± 1.05 (sample S12) to 33.97 ± 0.8 (sample S11). There was no significant difference in zeta potential (P > 0.05) between the samples (S8, S10). We selected 5 ml of chloroform as the optimum volume of organic solvent for further preparation of liposomes because it gave us physically stable preparations with moderate EE%.

#### Different compound I concentration

Three different concentrations of compound I (5, 10, and 15 mg) were investigated. Other experimental parameters, such as the volume of organic solvent and lipid ratios, were kept constant. S10, S14, and S15 formulations (Table 3) were used with different compound I concentrations.

**Table 3:** Composition of liposomal preparations with different compound I concentrations.

Sample	PC:CL (mg)	CPA (mg)	MPA (mg)	Amount of compound I (mg)	Chloroform (ml)
S10	165: 10	15	5	5	5
S14	160: 10	15	5	10	5
S15	155: 10	15	5	15	5

**Figure 5:**
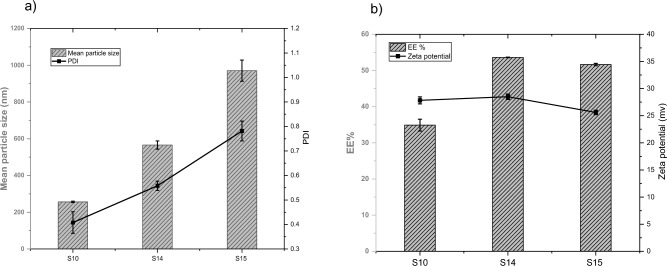
**(a, b**) The mean particle size, PDI and EE%, zeta potential of liposomal preparations with different Compound I concentrations (n = 3).

As shown in the Fig. 5a, the mean particle size was significantly increasing from 256.5 ± 3.8 nm (sample S10) to 970.5 ± 57.5 nm (sample S15) by increasing compound I concentration from 5 to 15 mg (P < 0.001). In addition, the PDI was significantly increasing as the compound I concentration was increased, and the differences in PDI between all populations (S10, S14, and S15) were significant (P < 0.01). Regarding the results of EE% and zeta potential shown in Fig. 5b**,** it was found that the EE% was significantly increased from 34.9 ± 1.65 to 53.62 ± 0.09 when compound I concentration was increased from 5 mg (sample S10) to 10 mg (sample S14), (P < 0.001). However, increasing compound I concentration to 15 mg (sample S15) did not affect increasing the EE%. This may be attributed to the saturation of liposome lipid bilayers, which inhibit more drug entrapment. Zeta potential ranged from 25.6 ± 0.4 mv to 28.5 ± 0.5 mv. The zeta potential was not significantly different between S10 and S14 (P > 0.05). Concentration of 10 mg of compound I was selected for further liposomal preparations as it showed the highest EE%, while increasing concentration above 10 mg did not increase EE%.

#### Type and concentration of steric stabilizing agent

Tween 80 and methyl ester of palmitic acid were evaluated as steric stabilizing agents. Samples that are nominated S, were prepared using methyl ester of palmitic acid (samples S1–S15), and the samples that were nominated T were prepared using Tween 80 as a steric stabilizing agent. Table 4 shows the composition of liposomal preparations prepared with different concentrations of Tween 80.

**Table 4:** Composition of liposomal preparations with different concentrations of Tween 80.

Sample	Final conc. of tween 80 (% w/v)	PC:CL (mg)	CPA (mg)	Compound I amount (mg)	Chloroform (ml)
T1	0.01	163: 10	15	10	5
T2	0.02	161: 10	15	10	5
T3	0.03	159: 10	15	10	5
T4	0.05	155: 10	15	10	5
T5	0.1	145: 10	15	10	5

**Figure 6:**
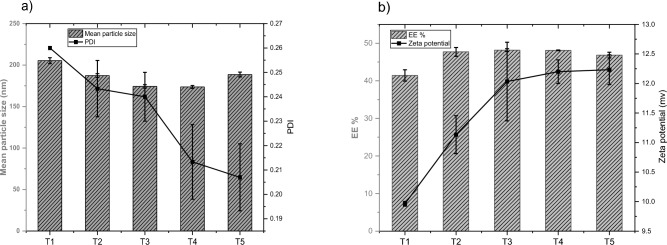
**(a, b)** The mean particle size, PDI and EE%, zeta potential of liposomal preparations prepared with different concentrations of Tween 80 (T1–T5) (n = 3).

As previously shown in Table 1, samples S1-S3, which were prepared using different concentrations of MPA (which was used as a steric stabilizing agent), were unstable. Owing to these results, we investigate the effect of Tween 80 as a steric stabilizing agent on the characteristics of liposome preparations. This study was a trial to decrease the mean particle size, enhance the PDI, and obtain more stable preparation. Even though we obtained liposomal preparation with good EE% (S14), which prepared using MPA as a steric stabilizing agent (Fig. 5b), the mean particle size and PDI were increased, in addition to the agglomeration occurred after storage for 1 day in the refrigerator. The characteristics of liposomal preparations prepared with different concentrations of Tween 80 are demonstrated in Fig. 6**.** From the results in Fig. 6a, it was found that the mean particle size decreased significantly from 205.3 ± 3.5 nm to 173.67 ± 1.68 nm when the concentration of Tween 80 increased from 0.01% w/v (sample T1) to 0.05% w/v (sample T4), (P < 0.05). However, increasing the concentration of Tween 80 to 0.1% w/v slightly increased the mean particle size of liposomes to 188.6 ± 2.95 nm. By comparing between mean particle size of samples with Tween 80 and MPA at the same conditions, we found that Tween 80 was able to decrease the particle size of liposomal preparations, which may be due to the surface activity properties of Tween 80. The PDI was decreased from 0.26 to 0.207 when the concentration of Tween 80 increased from 0.01 to 0.1, but the differences in PDI were not significant (P > 0.05). Concerning the EE%, it was observed that there were slight differences in the EE% between the samples (T1-T5). The mean EE% of samples (T1-T5) were about 46.45 ± 2.86 (Fig. 6b). This is different from the findings observed by Wang et al.^26^, who reported that increasing the concentration of Tween 80 leads to a reduction in EE%. The zeta potential ranged from 9.96 ± 0.04 mv to 12.23 ± 0.25 mv, which indicated that increasing the concentration of Tween 80 to 0.1 lead to increasing the zeta potential and more system stabilization. This is similar to the findings obtained by Shafie et al.^27^, who concluded that increasing the concentration of Tween 80 increases the zeta potential of the prepared betamethasone loaded nanoparticles.

#### Effect of different types of hydrating aqueous solutions on characteristics of liposomes

Four different aqueous solutions were used in the hydration of liposomes; phosphate buffer (pH 6.8), 1% sugar, distilled water, and 0.9% sodium chloride for injection. The influence of these types on the physical stability and appearance of liposomal suspension, the particle size, PDI, and zeta potential were studied and investigated. The aqueous solution that produced physically stable liposomal suspension with appropriate particle size and PDI was used for further biological studies. Table 5 demonstrates the composition of liposomes prepared using different aqueous solutions for hydration.

**Table 5:** Liposomal compositions with different types of aqueous solutions for hydration process

Sample	Final conc. of tween 80 (% w/v)	PC:CL (mg)	CPA (mg)	Aqueous solution for hydration
T4	0.05	163: 10	15	phosphate buffer (pH 6.8)
T6	0.05	161: 10	15	0.9% sodium chloride
T7	0.05	159: 10	15	distilled water
T8	0.05	155: 10	15	1% sugar
T8*	0.1	150: 10	10	1% sugar

**Figure 7:**
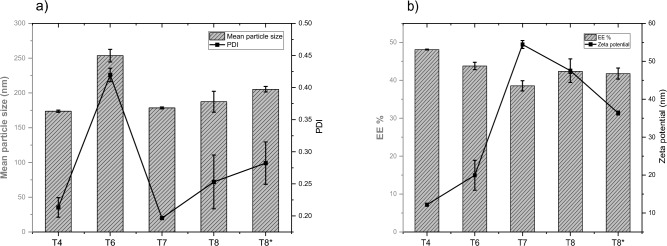
(**a, b**) The mean particle size, PDI and EE%, zeta potential of liposomal preparations (T4–T8*) prepared with different types of aqueous hydrating solutions (n = 3).

The type of aqueous solution used for hydration during liposomes preparations was found to have a great influence on the particle size, PDI, and zeta potential (Fig. 7). As shown in Fig. 7a, using 0.9% NaCl solution (sample T6) showed the highest particle size (253.57 nm ± 9.03) compared to using phosphate buffer (sample T4), distilled water (sample T7) and 1% sugar solution (sample T8) with mean particle sizes 173.67nm ± 1.68, 178.47 nm ± 1.39 and 187.33 nm ± 15.04 respectively. In addition, the maximum PDI was observed with sample T6 prepared using 0.9% NaCl solution during hydration compared to other samples (T4, T7, T8). On contrast, the minimum PDI was noted with sample T7 (PDI 0.197 ± 0.0021) when using distilled water as a hydrating solution. The EE % ranged from 38.54% ± 1.34 (sample T7) to 48.10% ± 0.0995 (sample T4). The major impact was observed with the zeta potential while using different aqueous solutions for hydration. The differences in the results of zeta potential were significant between all populations and each other (T4, T6, T7, T8, and T8*). The zeta potential value is a crucial aspect of a particle since it has an impact on both cell adhesion and particle stability. By interacting with negatively charged genes to penetrate biological membranes and reach target-specific areas in vivo, the positive surface charge of nanoparticles provides an electrostatic repulsive force against particle aggregation^28^. From Fig. 7b, it was observed that sample T7 has the highest zeta potential (54.4 mv ± 1.04), which was prepared using distilled water as hydrating aqueous solution, while sample T4 has the lowest zeta potential prepared with phosphate buffer. Using 1% sugar solution in sample T8 lead to reduction in zeta potential to + 47.53 mv ± 3.12. This finding may be due to more shielding of the charge by the addition of solutes in distilled water such as 0.9% NaCl or 1% sugar or in case of phosphate buffer. Developing these ions in the aqueous solution led to more charge shielding as indicated by a significant reduction in the zeta potential in these formulations (T4, T6, T8 and T8*). However, positively charged liposomes have a tendency to maintain the colloidal dispersion, resulting in vesicles with a smaller size and a narrow polydispersity index; increasing the positive charge has the potential to induce cytotoxicity^29^. There should be a balance between particle size, PDI, EE%, and surface charge of the formulations. In order to reduce the surface charge while maintaining mean particle size, PDI, and EE, we prepared sample T8* with more reduction in the concentration of CPA and an increase in Tween 80 concentration (see the composition Table 5). The zeta potential was observed to decrease to + 36.37 mv ± 0.49 with reasonable particle size, PDI, and EE%. By comparison of cytotoxicity of blank liposomal preparations similar to samples T7 and T8*, it was observed that the cell viability decreased with the blank sample possessing higher zeta potential (blank samples of T7 and T8*, results not shown). As reported by Shao et al.^28^, nanoparticles with positive zeta potentials caused significantly higher cytotoxic effects than nanoparticles with negative zeta potentials, and nanoparticles with large similar charges were more toxic. Consequently, we selected sample T8* for further biological studies instead of T7 because it has lower zeta potential (+ 36.37 mv ± 0.49), which is predicted to have lower cytotoxicity based on the results of cytotoxicity of their blanks similarly treated. Moreover, the particle size and EE% of sample T8* were 205.3 nm ± 3.94 and 41.78% ± 1.46 respectively, which were satisfactory and reasonable results.

### In vitro release study

**Figure 8:**
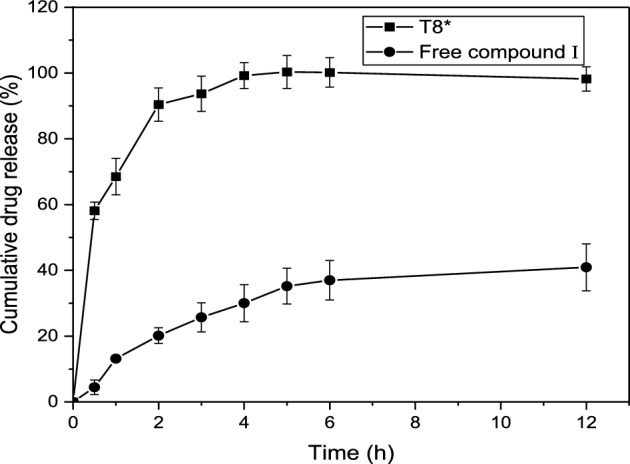
Cumulative drug release of sample T8* compared to free compound I in PBS (pH 6.8) (n = 3).

The results of in vitro release from the selected sample (T8*) compared to free compound I in PBS (pH 6.8) are demonstrated in Fig. 8. From the results, it is confirmed that the incorporation of compound I with liposomes significantly enhanced the drug release. This property might be helpful in improving the antitubercular efficacy of the liposomes since a greater amount of drug can be released at the target tissue at physiological pH. During the initial first hour of the release study, the liposomal sample T8* showed cumulative release of compound I of about 68.52% ± 5.5 while the release of free drug was found to be 13.15 ± 0.76. After 4 h of study, almost 99.2% ± 3.97 was released from T8* compared to about 30.0% ± 5.62 from pure compound I suspension. After 6 h of study, 36.99% ± 6 of the drug from free compound suspension 1 was released. According to these findings, compound I inclusion with liposomes considerably improved the drug release, which is expected to have a great impact on biological activity studies.

### Atomic force microscopy (AFM)

**Figure 9:**
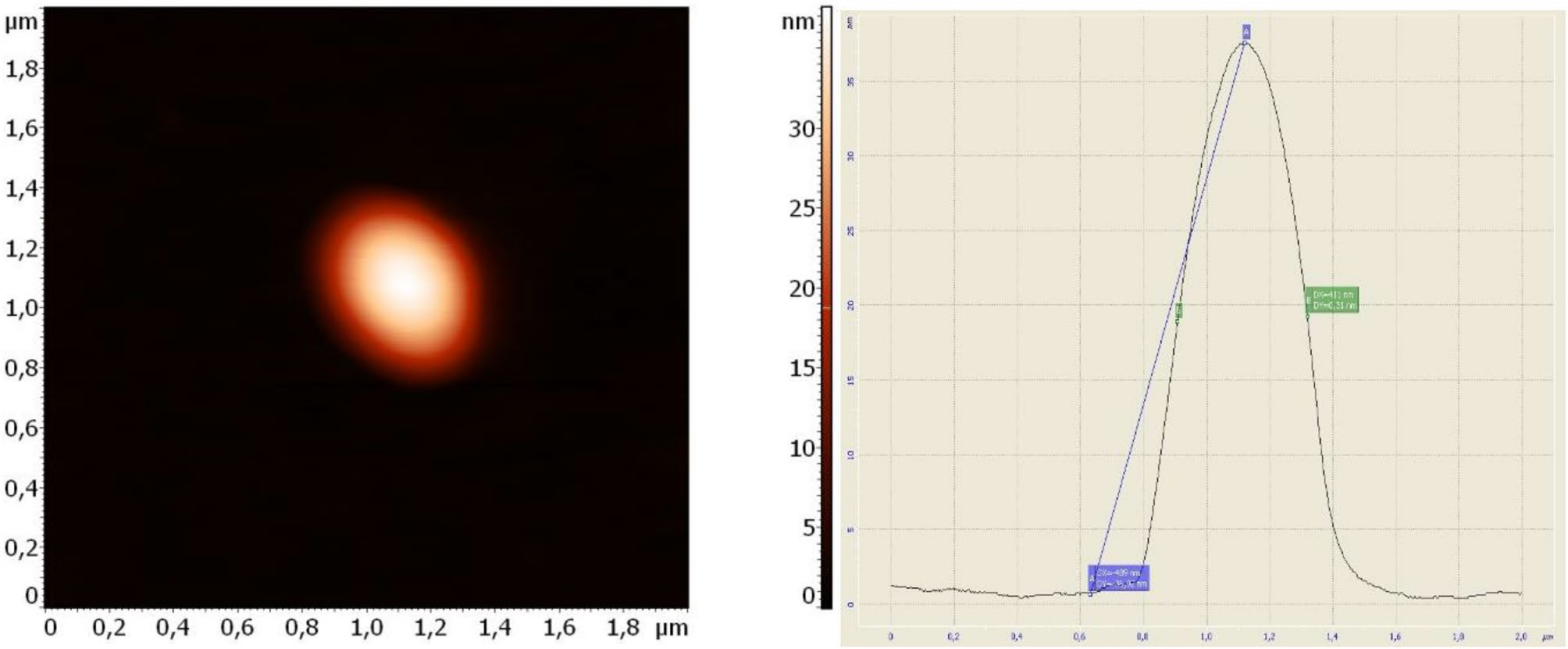
AFM image and size analysis of one particle of the selected formula T8*

Figure 9 demonstrates AFM image of one particle of the optimized formula T8*. From the figure, it is indicated that the particle is more compact and near spherical structure. The size was larger than that observed by DLS analysis (~ 411 nm), which may be due to the effect of AFM probe on the liposomal particles, thus may elongate them and lead to an increase in the particle diameter. These results are in agreement with the results obtained by Singh et al.^30^, who observed that the size of the solid lipid nanoparticles from AFM technique was much larger than the size observed by the Photon Correlation Spectroscopy technique.

### Biological studies

#### In vitro antitubercular activity and determination of MIC

The MIC of pure compound I, liposomal sample T8*, T8* with DMSO and empty liposomes are shown in Table 6. The concentrations of pure compound I and liposomal sample T8* that inhibit the growth of *M. tuberculosis* H_37_R_v_ were 0.94 and 1.88 μg/ml for both. The sample T8* with DMSO (conc. 1.5% v/v) inhibited the growth of *M. tuberculosis* (strain H_37_R_v_) at a concentration of 1.88 μg/ml. The empty liposomes showed no antimycobacterial activity at a concentration of 15 and 7.5 μg/ml.

**Table 6:** The MIC determination of pure compound I, selected liposomal sample T8* and T8* with DMSO against M. tuberculosis (strain H37Rv) by resazurin microtiter plate assay.

Sample	MIC (μg/ml)	Note
Pure compound I	0.94–1.88	DMSO conc. 1.5% v/v
T8*	0.94–1.88	–
T8* with DMSO	1.88	DMSO conc. 1.5% v/v
Isoniazid	0.03	–

#### Cytotoxicity study

**Figure 10:**
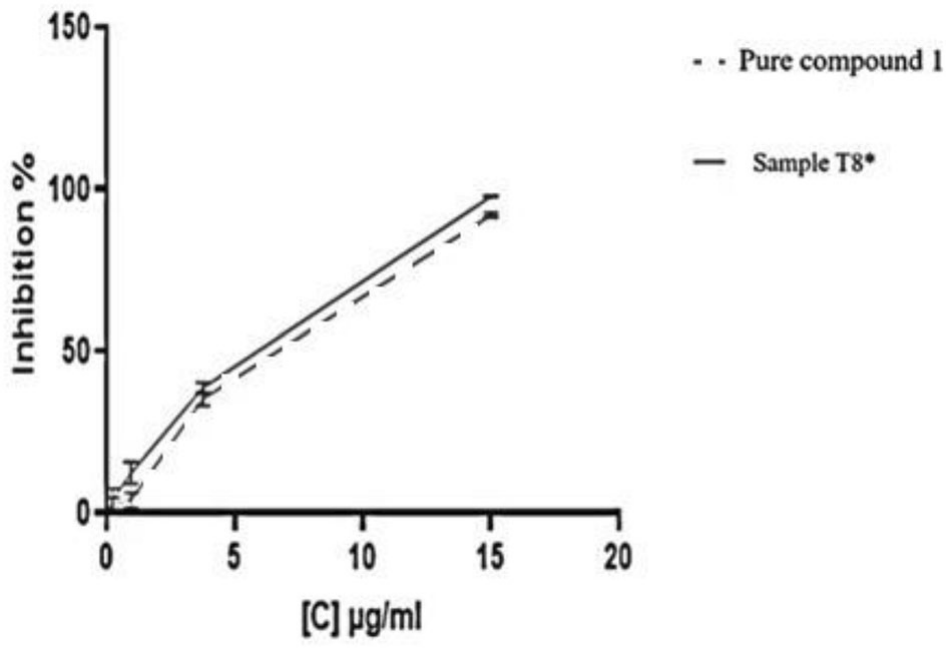
Percent inhibition of cell growth versus drug concentration.

Curves of dependence of cell viability on the concentration of the test compound were built. For the plate with pure compound I, 100% viability was taken to be the optical density values ​​obtained in the wells, where the cells were incubated in a nutrient medium with a DMSO concentration of 0.5% vol. For the plate with the liposomal form of compound I (sample T8*) and for the plate with "empty" liposomes, the values of optical density were obtained in the wells, where the cells were incubated in a nutrient medium without the addition of foreign compounds, were taken as 100% viability. The cytotoxic effect of pure compound I and liposomes loaded with compound I (sample T8*) by MTT assay is illustrated in Fig. 10, which revealed that pure compound I and liposomes loaded with compound I (sample T8*) were not cytotoxic at a concentrations less than 3.75 μg/ml. It was observed that cytotoxicity is a concentration dependent effect. Increasing the concentration of pure compound I or liposomes loaded with compound I (sample T8*) above 3.75 μg/ml leads to an increase in the percent inhibition of cell growth and hence, more toxic effect compared to free compound I. The liposomal sample (T8*) induced higher toxicity at a concentration of 15 μg/ml. The higher toxicity of the liposomes could be explained by the higher amounts of the drug released inside the cells. This is in accordance with Nguyena et al., who reported that the higher toxicity of both coated and uncoated liposomes compared to free docetaxel may be due to the higher cellular uptake following specific binding of hyaluronic acid from the liposomal surfaces to cell receptors, thereby releasing higher amounts of the drug inside the cells^31^. The IC50 values were calculated as the concentration of the compound causing a 50% reduction in cell viability for the pure compound I and sample T8* are shown in Table 7. No statistically significant differences were found when comparing the IC50 values of the pure compound I and sample T8* using the nonparametric Mann–Whitney test. Empty liposomes did not show considerable cytotoxicity at a concentration equivalent to the concentration of the liposomal form loaded with compound I of 3.75 μg/ml.

**Table 7:** IC50 values for pure compound I and sample T8* (n = 3).

Sample	IC50 (μg/ml)
Compound I	5.60 ± 1.60
T8*	5.37 ± 0.78

## Conclusion and future studies

In the current study, positively charged liposomal formulations loaded with compound I were developed and optimized. The influence of different formulation parameters such as compound I concentration, type of steric stabilizer, organic solvent, and aqueous solutions were accurately investigated. Sample T8* was physically stable and showed a mean particle size of about 205.3 nm ± 3.94, PDI 0.28 ± 0.033, EE % approximately 41.77% ± 1.456 and zeta potential 36.37 mv ± 0.49. The results of the in vitro release study indicated that the release of compound I was enhanced from liposomal sample T8* compared to the release of the free compound I and was selected for biological studies. The Free compound I and sample T8* showed antimycobacterial activity against *M. tuberculosis* *H*_*37*_*R*_*v*_ with MIC of about 0.94–1.88 μg/ml. The differences in the IC50 values determined from MTT assay for T8* and pure compound I were not significant (P > 0.05). Future studies are needed, such as using liposomes coated with different concentrations of polymers and finding additives that enhance their uptake by macrophages and to be safe for pulmonary administration. In conclusion, liposomes loaded with compound I may be a promising system for the pulmonary delivery of new antitubercular drugs.

### Supplementary Information

Supplementary Information.

## Data Availability

The datasets analyzed during the current study are available from the corresponding author on reasonable request.
