# Author Correction: Optimization, characterization, and cytotoxicity studies of novel anti-tubercular agent-loaded liposomal vesicles

**DOI:** 10.1038/s41598-024-52283-1

**Published:** 2024-01-25

**Authors:** Manar M. Obiedallah, Maxim A. Mironov, Danila V. Belyaev, Antoaneta Ene, Diana V. Vakhrusheva, Svetlana Yu. Krasnoborova, Sergey Y. Bershitsky, Daniil V. Shchepkin, Artem S. Minin, Rashida I. Ishmetova, Nina K. Ignatenko, Svetlana G. Tolshchina, Olga V. Fedorova, Gennady L. Rusinov

**Affiliations:** 1 Institute of Chemical Technology, Ural Federal University, Yekaterinburg, Russia; 2 Department of Pharmaceutics, Faculty of Pharmacy, Assiut University, Assiut, 71526 Egypt; 3 I. Postovsky Institute of Organic Synthesis, Ural Branch of the Russian Academy of Sciences, S. Kovalevskaya Str. 22, Yekaterinburg, 620108 Russia; 4 National Medical Research Center of Phthisiopulmonology and Infectious Diseases, 22 Parts’ezda St., 50, Yekaterinburg, 620039 Russia; 5 INPOLDE Research Center, Department of Chemistry, Physics and Environment, Faculty of Sciences and Environment, Dunarea de Jos University of Galati, 47 Domneasca Street, 800008 Galati, Romania; 6 Institute of Immunology and Physiology, Ural Branch of Russian Academy of Sciences, Yekaterinburg, 620049 Russia; 7 Institute of Natural Sciences and Mathematics, Ural Federal University, Yekaterinburg, Russia; 8 M.N. Mikheev Institute of Metal Physics of the Ural Branch of the Russian Academy of Sciences, S.Kovalevskaya St. 18, Yekaterinburg, 620108 Russia

Correction to: *Scientific Reports* 10.1038/s41598-023-49576-2, published online 04 January 2024

The original version of this Article omitted an affiliation for Manar M. Obiedallah. The correct affiliations are listed below.

Institute of Chemical Technology, Ural Federal University, Yekaterinburg, Russia.

Department of Pharmaceutics, Faculty of Pharmacy, Assiut University, Assiut 71526, Egypt.

The original Article has been corrected.

