# Supplementary material for: Optimization, characterization, and cytotoxicity studies of novel anti-tubercular agent-loaded liposomal vesicles
Source: Sci Rep. 2024 Jan 4;14:524. doi: 10.1038/s41598-023-49576-2 (PMC10766644; doi:10.1038/s41598-023-49576-2)
Supplement: Supplementary file 1 — Supplementary Information. [file 41598_2023_49576_MOESM1_ESM.docx]

**Supplementary Materials for:**

**Novel Antitubercular Agent-Loaded Liposomal Vesicles: Optimization, Characterization, and Cytotoxicity Studies**

Manar M. Obiedallah ^1,2*^; Maxim A. Mironov ^1*^; Danila V. Belyaev ^3,4^; Antoaneta Ene ^5*^; Diana V.Vakhrusheva ^4^; Svetlana Yu. Krasnoborova ^4^; Sergey Y. Bershitsky ^6^; Daniil V. Shchepkin ^7^; Artem S. Minin ^8^; Rashida I. Ishmetova ^3^; Nina K. Ignatenko ^3^; Svetlana G. Tolshchina ^3^; Olga V. Fedorova ^3^; Gennady L. Rusinov ^1,3^.

^1^ Institute of Сhemical Technology, Ural Federal University, Ekaterinburg, Russia.

^2^ Department of Pharmaceutics, Faculty of Pharmacy, Assiut University, Assiut 71526, Egypt

^3^ I. Postovsky Institute of Organic Synthesis, Ural Branch of the Russian Academy of Sciences, S. Kovalevskaya Str. 22, Ekaterinburg 620108, Russia

^4^ National Medical Research Center of Phthisiopulmonology and Infectious Diseases, 22 Parts’ezda St., 50, Ekaterinburg, 620039, Russia

^5^ INPOLDE Research Center, Department of Chemistry, Physics and Environment, Faculty of Sciences and Environment, Dunarea de Jos University of Galati, 47 Domneasca Street, 800008 Galati, Romania

^6^ Institute of Immunology and Physiology, Ural Branch of Russian Academy of Sciences, Yekaterinburg 620049, Russia

^7^ Institute of Natural Sciences and Mathematics, Ural Federal University, Ekaterinburg, Russia.

^8^ M.N. Mikheev Institute of Metal Physics of the Ural Branch of the Russian Academy of Sciences, Ekaterinburg, 620108, S.Kovalevskaya st. 18, Russian Federation.

*Corresponding author:* [*m.mh.obid@gmail.com*](mailto:m.mh.obid@gmail.com) *(Manar M. Obiedallah)*

[*m.a.mironov@urfu.ru*](mailto:m.a.mironov@urfu.ru) *(Maxim A. Mironov)*

[*Antoaneta.Ene@ugal.ro*](mailto:Antoaneta.Ene@ugal.ro) *(Antoaneta Ene)*


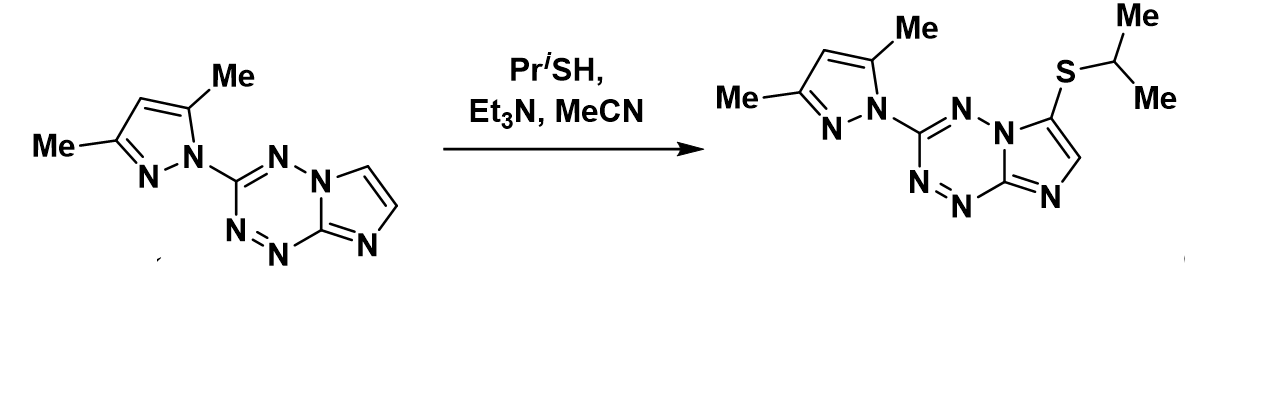

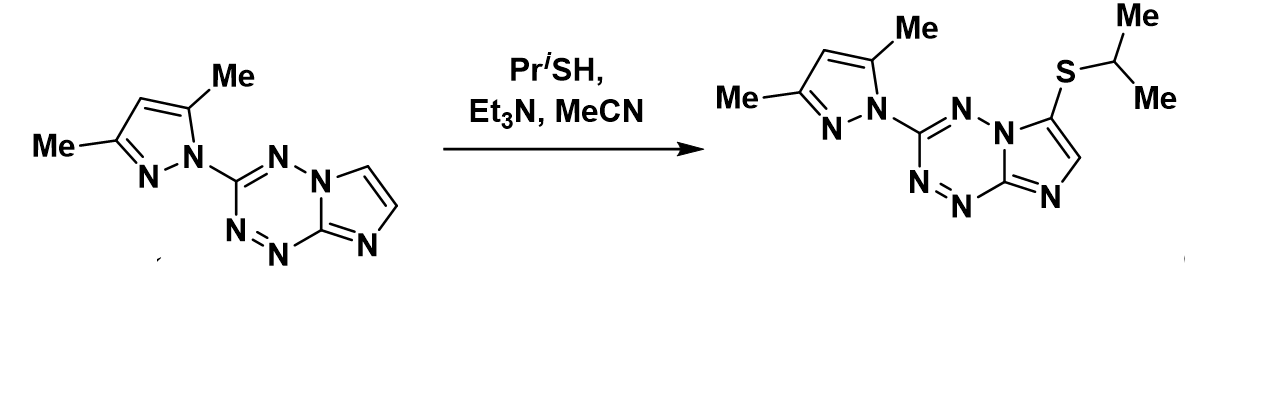


Compound Ⅰ

**Fig. M1.** Synthesis of compound Ⅰ (3-(3,5-dimethylpyrazole-1-yl)-6- (isopropylthio)imidazo[1,2-b][1,2,4,5]tetrazine (I)


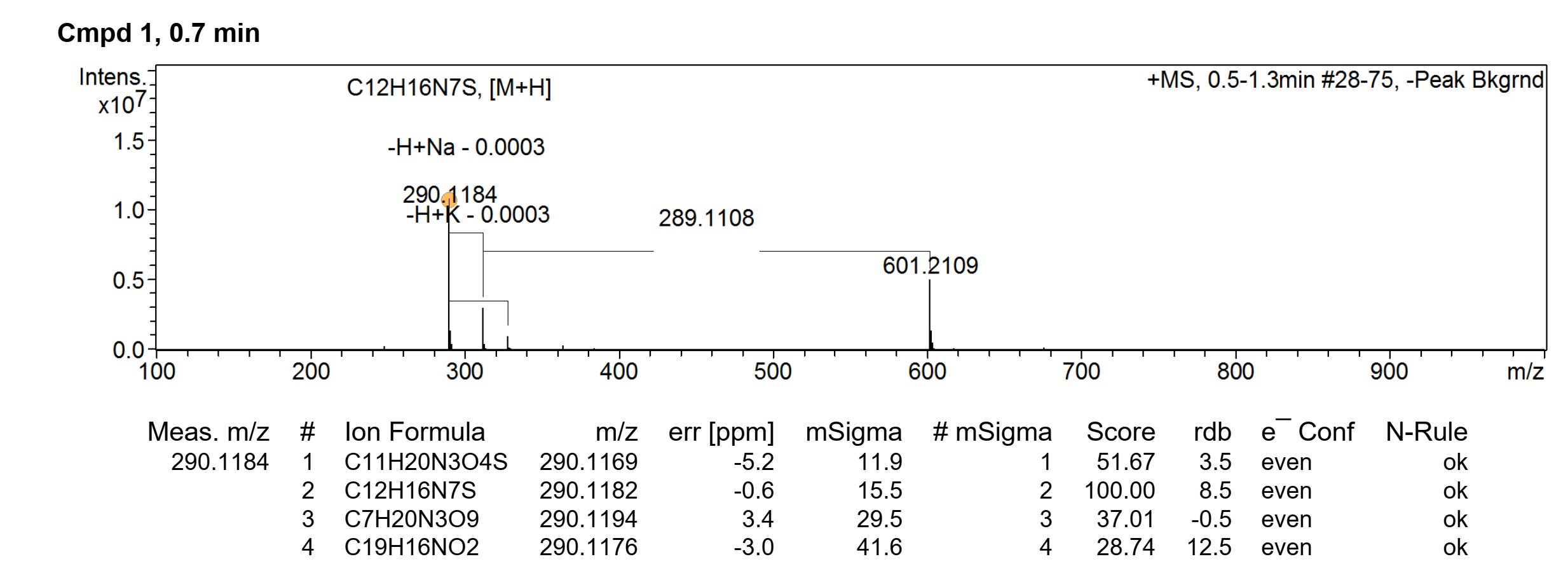


**Fig. M2.** HRMS of compound Ⅰ in acetonitrile

**Fig. M3.** GC chromatogram of compound Ⅰ in acetonitrile

**Fig. M4.** IR spectrum of compound Ⅰ
